# NLRP3, NLRC4 and NLRC5 Gene Polymorphisms Associate with Susceptibility of Pulmonary Aspergillosis in Non-Neutropenic Patients

**DOI:** 10.3390/jcm11071870

**Published:** 2022-03-28

**Authors:** Jinjin Zhong, Lulu Liu, Yajie Lu, Yu Gu, Jiangnan Zhao, Bilin Chen, Wei Zhou, Xin Su

**Affiliations:** Department of Respiratory and Critical Care Medicine, Jinling Hospital, Medical School of Nanjing University, Nanjing 210002, China; dg20350104@smail.nju.edu.cn (J.Z.); dg1735068@smail.nju.edu.cn (L.L.); mf20350132@smail.nju.edu.cn (Y.L.); gulnurjl@163.com (Y.G.); dg1735035@smail.nju.edu.cn (J.Z.); dg1835055@smail.nju.edu.cn (B.C.); mg1435144@smail.nju.edu.cn (W.Z.)

**Keywords:** pulmonary aspergillosis, NLRP3, NLRC4, NLRC5, gene polymorphisms

## Abstract

Background: Non-neutropenic pulmonary aspergillosis is one of the most common and serious fungal infections. Previous studies have shown that single nucleotide polymorphisms (SNPs) of pattern recognition receptors genes are associated with susceptibility to aspergillosis. NOD-like receptors (NLRs) play an important role in the immunological response against fungal infection. In this study, we investigated the relationship between polymorphisms of three NLRs and susceptibility to pulmonary aspergillosis disease in non-neutropenic patients. Methods: We included 73 patients with proven pulmonary aspergillosis and 103 healthy controls. A total of sixteen SNPs in the NLRP3, NLRC4, and NLRC5 genes were detected by PCR-direct sequencing. Then, we evaluated the association between these polymorphisms and susceptibility to aspergillosis. Results: Fifteen SNPs were consistent with Hardy–Weinberg equilibrium except for NLRP3 rs7525979. A total of eight SNPs (NLRP3 rs3806265, NLRC4 rs212704 and NLRC5 rs1684579, rs12598522, rs3995817, rs3995818, rs34531240, rs28438857) were observed an association with susceptibility of pulmonary aspergillosis. The CC homozygote of NLRP3 rs3806265, TT homozygote of NLRC5 rs1684579 and T allele of NLRC5 rs12598522 were associated with a higher risk of aspergillosis while TT homozygote of NLRC4 rs212704 was associated with a lower risk of aspergillosis. Especially in the invasive pulmonary aspergillosis subgroup, the TT homozygote of NLRC5 rs1684579 and rs3995817, the CC homozygote of NLRC5 rs34531240 and rs28438857, GG homozygote of NLRC5 rs3995818, the C allele and CC homozygote of NLRP3 rs3806265 were associated with higher susceptibility. Conclusions: This study showed an association between polymorphisms of NLRP3, NLRC4, and NLRC5 and susceptibility to pulmonary aspergillosis for the first time. Further investigations in larger populations are needed, and functional studies are also required to investigate the function of these NLRs in aspergillosis, as well as other fungal infection diseases.

## 1. Introduction

*Aspergillus* is a saprotrophic fungus which spreads by asexual conidia. The airborne *Aspergillus* conidia can be inhaled into the respiratory tract and lungs of humans, causing different types of diseases, including invasive pulmonary aspergillosis (IPA), chronic pulmonary aspergillosis (CPA), and allergic bronchial pulmonary aspergillosis (ABPA) [1,2,3]. Since the late 1990s, aspergillosis has actually proved to be the most common invasive pulmonary fungal infection. Moreover, it has also become the most expensive fungal disease because of its prevalence and costly treatments [4].

Pattern recognition receptors (PRRs), including Toll-like receptors, RIG-I-like receptors, NOD-like receptors, and C-type lectin receptors, play an important role in host immunity against *Aspergillus*; thus, the genetic defects in PRRs may lead to susceptibility to aspergillosis [5,6,7]. Polymorphisms in Toll-like receptors such as TLR4, C-type lectin receptors such as dectin-1, and other pattern recognition receptors such as Pentraxin 3 (PTX3) have been found to be associated with susceptibility to aspergillosis [8,9,10].

The NOD-like receptors make up an important family of PRRs. Many of them can bind with apoptosis-associated speck-like protein containing a CARD(ASC) and caspase-1 to form inflammasome, such as NLRP3, NLRC4, NLRC5 inflammasome, and so on [11]. In an aspergillosis mouse model, several kinds of NLRs increased in the infected lungs, including NLRP3, NLRC4, and NLRC5, but their exact functions remain to be explored [12]. NLRP3 is the most well-characterized and most well-studied inflammasome sensor molecule. When the NLRP3 inflammasome is activated, Pro-Caspase-1 is cleaved, which leads to the release of proinflammatory cytokines like IL-1β and IL-18, as well as pyroptotic cell death [13,14]. Generally, NLRP3 is known to contribute to antifungal immunity and help control infection [5]. For instance, a GAG-deficient *Aspergillus* mutant, which failed to elicit protective NLRP3 inflammasome activation, exhibited enhanced virulence [15]. Likewise, the mice lacking AIM2 and NLRP3 were susceptible to *Aspergillus* infection [16]. Similarly, the NLRC4 inflammasome has been also found to protect mucosal barriers such as the lung, stomach, and intestine from invading pathogens [17]. NLRC5 is the largest one in the NLR family and works as the master transcriptional regulator of MHC class I and related genes [18,19]. Therefore, after infection, NLRC5 knockout mice showed increased bacterial load and impaired clearance of viruses due to strongly impaired MHCI-mediated CD8+ T cell activation [20,21,22]. However, the role of NLRC4 and NLRC5 in *Aspergillus* infection is still unknown. Since innate immunity and adaptive immune responses are all important parts for host defense against *Aspergillus*, we assume that they are probably involved in the host immune response in aspergillosis, as well as in other PRRs. 

A few previous studies have shown the polymorphisms of NLRs genes affected the susceptibility of *Aspergillus* infection or colonization. Among transplant recipients after hematopoietic stem cell transplantation (HSCT), P268S (rs2066842) in NOD2 of the donors was associated with an increased risk of invasive aspergillosis [23]. For patients with cystic fibrosis, significant associations were found between *A.fumigatus* colonization and polymorphisms of NLRC4, including the haplotype ACTT (rs212704 rs455060 rs7562653 rs385076) and GG genotype of rs212704 [24]. In this study, we investigated 16 SNPs in NLRP3, NLRC4, and NLRC5 genes among the southeastern Han Chinese population and analyzed the relationships between these SNPs and susceptibility of pulmonary aspergillosis in non-neutropenic patients.

## 2. Materials and Methods

### 2.1. Study Population

This study included 73 pulmonary aspergillosis patients treated at Jinling Hospital from June 2016 to December 2019. The control group consisted of 103 healthy people undergoing physical examination. According to the updated IDSA guideline criteria and EORTC/MSGERC criteria, these aspergillosis patients were diagnosed as 30 IPA, 27 CPA (including chronic cavitary pulmonary aspergillosis, CCPA and aspergilloma), and 16 ABPA [25,26] (Table 1). Since the study subjects were non-neutropenia patients, we excluded patients who had previously undergone organ transplantation or chemotherapy.

### 2.2. Selection of SNPs and Genotyping

The single nucleotide polymorphisms of NLRP3 (rs3806265, rs7525979, rs35829419, rs10754558) and NLRC4 (rs12989936, rs212704, rs7562653, rs479333, rs385076) were selected from previous literature. Seven SNPs of NLRC5 (rs12598522, rs34531240, rs28438857, rs3995818, rs3995817, rs1684579, rs3751705) were selected based on information from the NCBI GenBank, dbSNP, and HapMap databases, with the minimum allele frequency set at 5% and r2 at 0.8. These SNPs were located within the coding region, 5′ untranslated region (UTR), or 3′UTR that may possibly influence protein synthesis and gene transcription. Peripheral blood (1 mL) was collected in an EDTA tube from each subject.

Genomic DNA was extracted from the whole blood using the QIAamp DNA Blood Mini Kit (Qiagen, Berlin, Germany) according to the manufacturer’s instructions and then stored in a −80 °C freezer. Primers were designed using Primer Premier 5.0 (Premier Biosoft International, Palo Alto, CA, USA). PCR amplification was performed in Eppendorf PRO PCR System (Hamburg, Germany). All SNPs were genotyped by ABI Prism 377 Sequence Detection System (Applied Biosystems, Foster City, CA, USA) with technical support from the Shanghai Genesky Biotechnology Company (Shanghai, China). DNA sequences were read by Chromas 2.3 software (Technelysium Pty Ltd., Tewantin, Australia). Negative controls were included in each plate for accuracy.

### 2.3. Statistics

Each SNP was tested for Hardy–Weinberg equilibrium (HWE) in the healthy control group using the chi-square (χ^2^) test. Statistical comparison was performed by independent Student’s *t*-test or one-way analysis of variance. Differences among SNPs were evaluated using Pearson’s χ^2^ or Fisher’s exact test. The strength of association between polymorphisms and the risk of aspergillosis was evaluated by odds ratio (OR) and 95% confidence interval (CI). HWE, linkage disequilibrium, and haplotype analysis were analyzed by HaploView [27,28]. Statistical analysis was performed by the SPSS 24.0 and the GraphPad Prism 7. All tests were considered significant with a *p* value of <0.05.

## 3. Results

### 3.1. Characteristics of the Patient Population

We enrolled 73 patients with pulmonary aspergillosis and 103 healthy controls. Healthy controls were used to compare the frequency of mutations between the patient group and the general population, and there were no significant differences in age (58.48 ± 1.63 vs. 55.25 ± 1.038, *p* = 0.0819) or sex (male/female: 41/32 vs. 64/39, *p* = 0.4263) between the case and control groups. Table 1 summarizes the main characteristics of the study population. The case group included 20 (27.40%) patients with hypertension and 12 (16.44%) with diabetes, 11 (15.07%) with COPD, 5 (6.85%) with asthma, 23 (31.51%) with bronchiectasis, and 20 (27.40%) with tuberculosis. In addition, 21 (28.77%) in the case group had a history of smoking and 13 (17.81%) had a history of steroid treatment prior to onset.

### 3.2. Hardy-Weinberg Equilibrium Analysis and Minor Allele Frequencies of SNPs

We analyzed HWE and minor allele frequencies (MAF) of the 16 SNPs. All but NLRP3 rs7525979 were consistent with HWE in the control group (*p* > 0.05). No allele was detected at NLRP3 rs35829419 in either control group or case group. MAF of the other SNPs were >5%. So, we excluded these two SNPs in the following statistical analysis. Other information regarding these SNPs is shown in Table 2.

### 3.3. Association of NLRs Variants with Pulmonary Aspergillosis

First, we evaluated the association of 14 single nucleotide polymorphisms with pulmonary aspergillosis risk among all patients and healthy controls (Table 3). A total of 4 SNPs (NLRP3 rs3806265, NLRC4 rs212704, NLRC5 rs1684579, and rs12598522) were observed an association with aspergillosis risk. Genotype difference in NLRP3 rs3806265 between the case and control groups was statistically significant (*p* = 0.0451) and the CC homozygote of rs3806265 was associated with a higher risk of aspergillosis (*p* = 0.0130; OR = 2.567, 95% CI: 1.239 to 5.255). For the NLRC4 rs212704, the TT homozygote was associated with a lower risk of aspergillosis (*p* = 0.0447; OR = 0.4468, 95%CI: 0.2071 to 0.959) while for the NLRC5 rs1684579 it was opposite (*p* = 0.0261; OR = 2.066, 95%CI: 1.085 to 4.018). Furthermore, the T allele of NLRC5 rs12598522 was more frequent in aspergillosis patients than healthy controls (*p* = 0.0305; OR = 1.601, 95%CI: 1.048 to 2.47). The other SNPs did not show any association with aspergillosis risk (Supplementary Table S1).

### 3.4. Association of NLRs Variants with Different Kinds of Aspergillosis

Next, we divided the patients into non-ABPA subgroup and ABPA subgroup according to whether the pathological process was mainly an inflammatory or allergic response and then compared each of the two subgroups with healthy controls (Table 4). In the non-ABPA group, gene polymorphism of the rs3806265 in NLRP3 and rs12598522, rs1684579 in NLRC5 showed a significant association with susceptibility. Similar to the entire group analysis, genotype difference in NLRP3 rs3806265 was statistically significant (*p* = 0.0298) and the CC homozygote was associated with a higher risk (*p* = 0.0129; OR = 2.702, 95%CI: 1.172 to 5.924), as well as the T allele of NLRC5 rs12598522 (*p* = 0.0419; OR = 1.612, 95%CI: 1.012 to 2.529). For the NLRC5 rs1684579, there was also a significant genotype difference (*p* = 0.0125) and the TT homozygote (*p* = 0.0044; OR = 2.665, 95%CI: 1.371 to 5.292), as well as T allele (*p* = 0.0471; OR = 1.614, 95%CI: 0.9938 to 2.624) were significantly associated with a higher risk of *Aspergillus* infection. However, in the ABPA subgroup, there was no SNP associated with susceptibility (Supplementary Table S2).

Finally, we divided the non-ABPA group into IPA subgroup and CPA subgroup and compared them with control group (Table 5). It was worth noting that we found rs3806265 in NLRP3 and rs34531240, rs28438857, rs3995818, rs3995817, rs1684579 in NLRC5 were all associated with IPA risk. The CC homozygote (*p* = 0.0004; OR = 4.861, 95%CI: 2.007 to 11.9) and C allele (*p* = 0.0117; OR = 2.115, 95%CI: 1.196 to 3.741) of NLRP3 rs3806265, as well as TT homozygote of NLRC5 rs1684579 (*p* = 0.0036; OR = 3.385, 95%CI: 1.47 to 8.101) were associated with a higher risk of IPA and the genotype differences of them were significant between IPA patients and controls (*p* = 0.0017; *p* = 0092). The CC homozygote of rs34531240 and rs28438857, GG homozygote of rs3995818, TT homozygote of rs3995817 in NLRC5 were all more frequent in IPA patients than controls (*p* = 0.0420; OR = 2.386, 95%CI: 0.9749 to 5.567). However, in the ABPA subgroup, there was no SNP associated with susceptibility (Supplementary Table S3).

### 3.5. Linkage Disequilibrium and Haplotype Analyses

**Figure 1:**
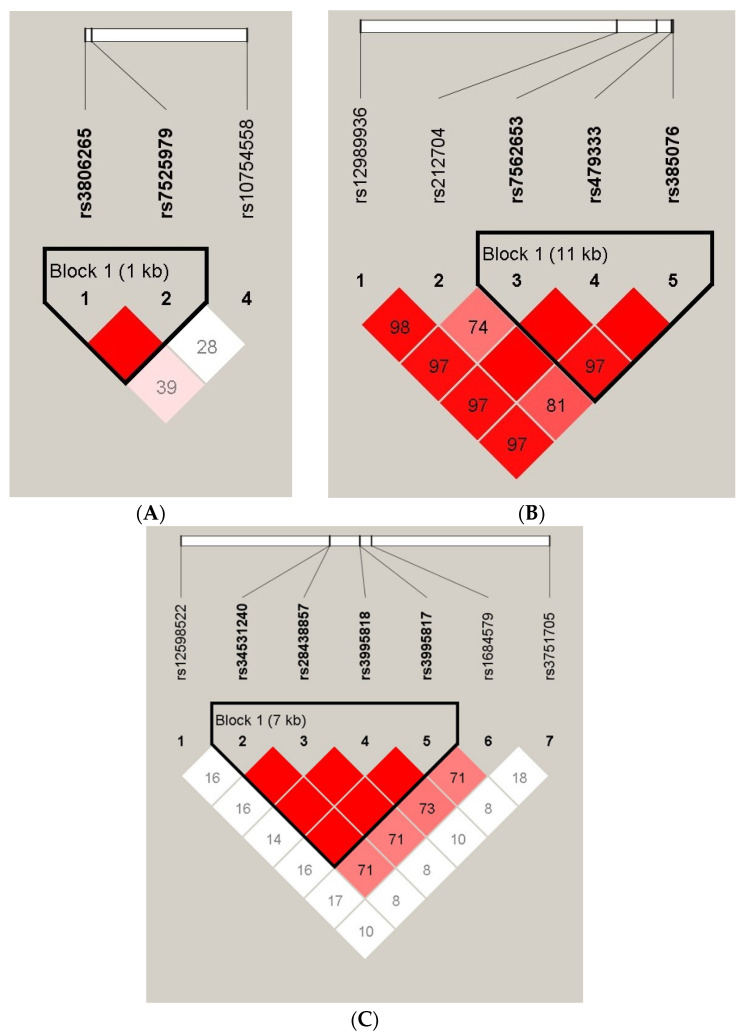
A linkage disequilibrium (LD) plot of NLR SNPs for all aspergillosis patients and controls. The D’ value of each SNP pair is represented as a percentage and shown in the square (D’ ≥ 0.99 not shown). Red squares indicate strong pairwise linkage disequilibrium. (**A**) The two NLRP3 SNPs (rs3806265, rs7525979) constitute a haplotype block spanning 1 kb of the NLRP3 gene with an extremely high pair-wise D′ ≥ 0.99. (**B**) The three NLRC4 SNPs (rs385076, rs47933, rs7562653) constitute a haplotype block spanning 11 kb of the NLRC4 gene (**C**) The four NLRC5 SNPs (rs34531240, rs28438857, rs3995818, rs3995817) constitute a haplotype block spanning 7 kb of the NLRC5 gene with an extremely high pair-wise D′ ≥ 0.99.

Linkage disequilibrium (LD) analysis showed that rs3806265, rs7525979 in NLRP3, rs385076, rs47933, rs7562653 in NLRC4, rs34531240, rs28438857, rs3995817, rs3995818 in NLRC5 were in high LD (D′ > 95) (Figure 1). In the NLRP3 rs3806265, rs7525979 block, haplotype TC showed an association with a decreased risk of IPA (*p* = 0.0117) while haplotype CC was more frequent among IPA patients (*p* = 0.0136) (Table 6).

## 4. Discussion

The aim of this study was to investigate the relationship between NLRP3, NLRC4, and NLRC5 gene polymorphisms and susceptibility to pulmonary aspergillosis in non-neutropenic patients among the Chinese population. For the first time, we found rs3806265 in NLRP3, rs212704 in NLRC4 and rs12598522, rs34531240, rs28438857, rs3995818, rs3995817, rs1684579 in NLRC5 were associated with pulmonary aspergillosis in non-neutropenic patients. 

The occurrence and development of aspergillosis are closely related to the host’s immune status. Therefore, polymorphisms of many immune-related genes are associated with susceptibility to aspergillosis, such as tumor necrosis factor receptor 1 (TNFR1), TLR1/4/5/6, Dectin-1, DC-SIGN, IL-8, IL-10, IL-12, IL-4R, IFN-γ, IRF4 and so on [8,9,10,29,30,31,32,33,34,35]. Decades ago, pulmonary aspergillosis (PA) often occurred in immunocompromised patients, in particular among hematopoietic stem cell transplants (HSCT) and patients with hematological malignancies. However, there is a rising incidence of pulmonary aspergillosis in non-neutropenic patients during recent years [36,37]. These previous studies were mostly based on patients with severe immunodeficiency, and there were few studies focusing on aspergillosis patients with non-severe immune deficiency, so our study selected non-neutropenic patients as the research object. 

NLR proteins are central mediators of microbial sensing with diverse functions, and they play an important role in the host antimicrobial immune responses including anti-*Aspergillus* response [38]. NLRs are usually composed of a tripartite structure, including an N-terminal effector domain, a central NACHT domain containing the nucleotide binding domain (NBD) for self-oligomerization and C-terminal leucine-rich repeats (LRRs) for recognizing PAMPs or DAMPs [39]. As for NLRs, previous studies have shown that rs2066842 in NOD2 of donors was associated with the risk of invasive aspergillosis after hematopoietic stem-cell transplantation, while rs212704 and ACTT (rs212704 rs455060 rs7562653 rs385076) in NLRC4 were associated with *A. fumigatus* colonization in cystic fibrosis patients [23,24]. In our study, we found the TT homozygote of rs212704 in NLRC4 and C allele of rs12598522 in NLRC5 was associated with a lower risk of aspergillosis. On the contrary, CC homozygote of rs3806265 in NLRP3 and TT homozygote of rs1684579 in NLRC5 was associated with a high risk of aspergillosis, especially of IPA. Besides, in the IPA subgroup, the CC homozygote of rs34531240 and rs28438857, GG homozygote of rs3995818, TT homozygote of rs3995817 in NLRC5 was more frequent than controls.

The polymorphism of rs3806265 in NLRP3 is associated with several kinds of diseases, such as myasthenia gravis (MG), psoriasis, recurrent aphthous stomatitis (RAS) and relapsing–remitting multiple sclerosis (RRMS) [40,41,42,43]. In the Iranian population, the C allele and CC homozygote of rs3806265 was more frequent in MG and RRMS patients than controls, whereas the T allele and TT homozygote were less frequent in RAS patients [40,41,42]. In the Chinese population, the T allele was associated with a higher risk of psoriasis and this locus might function as an enhancer in the immune-related system [43]. In our study, it was the opposite that C allele and CC homozygote were associated with a higher risk of IPA. This might be due to IPA occurring as a result of immune deficiency.

Previous research has found GG homozygote of rs212704 in NLRC4 was associated with *A.fumigatus* colonization in cystic fibrosis patients [24]. Our study showed that TT homozygote was associated with a decreased risk of aspergillosis, reflecting the role of NLRC4 in host defense against *Aspergillus* infection as well. Besides, C allele of rs212704 was associated with increased insulin and lower glucose levels while CC homozygote with a lower 2-h postprandial C-peptide level, suggesting that the polymorphism of NLRC4 may also relate to body metabolism [44,45].

The polymorphism of NLRC5 is also related to the susceptibility, severity, and prognosis of different kinds of diseases. In chronic periodontitis, the rs289723 in NLRC5 gene was associated with chronic slight and chronic localized periodontitis susceptibility and the AA genotype was correlated with increased risk of disease development [46]. It suggests that NLRC5 may play a promoting role in the insurgence of inflammation. So, it is reasonable that we found that the polymorphism of NLRC5 influenced the susceptibility to pulmonary aspergillosis, especially IPA. Some other studies reported that there was also an association between NLRC5 SNPs and the survival of colorectal and rectal cancer [47,48]. 

Our study demonstrated that the polymorphisms of NLRs (NLRP3, NLRC4, NLRC5) were associated with pulmonary aspergillosis risk, but there were still some limitations. First, it was a single-center study in southeastern China. So, it was hard to get a large case group, and our results might be influenced by geographic, ethnic, and genetic factors. Second, it was a retrospective study so we could not measure the NLRs expression or the levels of downstream inflammatory factors at the onset of aspergillosis. Likewise, we could only select healthy people as the control group instead of patients with the same underlying diseases and health condition. Finally, functional evaluations are needed to unveil the function of different SNPs of NLRs in the progression of aspergillosis. 

## 5. Conclusions

Our results identified the association of NLRP3, NLRC4, and NLRC5 genetic variation with the susceptibility of pulmonary aspergillosis for the first time. These NLRs are probably involved in host immune defense against *Aspergillus* infection. Their function and mechanism in aspergillosis have not yet been thoroughly studied. Our results can provide a reference for studies on the role of NLRs in aspergillosis and other fungal infections.

## Figures and Tables

**Table 1:** Demographic and clinical characteristics of the study population.

Characteristics	Case Group (*n* = 73)	Control Group (*n* = 103)	*p* Value
Age	58.48 ± 1.63	55.25 ± 1.038	n.s.
Gender (male/female)	41/32	64/39	n.s.
Ethnicity			n.s.
Han	73 (100)	103 (100)	
Serum albumin, g/L	32.23 ± 0.7314		
Comorbidities			
Hypertension	20 (27.40)		
Diabetes	12 (16.44)		
COPD	11 (15.07)		
Asthma	5 (6.85)		
Bronchiectasis	23 (31.51)		
Tuberculosis	20 (27.40)		
History of smoking	21 (28.77)		
Steroid treatment	13 (17.81)		
History of hepatitis infection	3 (4.11)		
Serum albumin < 30 g/L	27 (36.99)		
Classification			
IPA	30 (41.10)		
CPA	27 (36.99)		
ABPA	16 (21.92)		

**Table 2:** Information about NLRP3, NLRC4, and NLRC5 SNPs in this study.

Gene	SNP Number	Chromosome Position	Gene Location	MAF	HWE in Control Group
NLRP3	rs3806265	247586336	intron1	0.472	0.0628
	rs7525979	247587408	synon_exon3	0.188	0.0381
	rs35829419	247588858	nonsynon_exon3	0.0	1.0
	rs10754558	247612036	3′-UTR_exon10	0.469	0.269
NLRC4	rs12989936	32268586	3′-flanking	0.347	1.0
	rs212704	32450348	intron8	0.466	0.7296
	rs7562653	32478629	intron2	0.415	0.5904
	rs479333	32489158	intron1	0.344	1.0
	rs385076	32489851	intron1	0.392	0.9022
NLRC5	rs12598522	57022352	5′-flanking	0.5	0.8838
	rs34531240	57060340	synon_exon5	0.44	0.0882
	rs28438857	57060353	nonsynon_exon5	0.44	0.0882
	rs3995818	57068106	nonsynon_exon12	0.443	0.0882
	rs3995817	57068107	synon_exon12	0.44	0.0882
	rs1684579	57071113	synon_exon14	0.423	0.1275
	rs3751705	57116458	3′-UTR_exon48	0.327	0.5313

**Table 3:** Genotype distribution of NLRP3 rs3806265, NLRC4 rs212704, NLRC5 rs1684579, and rs12598522 in pulmonary aspergillosis cases and controls.

Gene	SNP	Model	Genotype	Case	Control	OR(95% CI)	*p* Value
NLRP3	rs3806265	Codominant	CC/CT/TT	21/35/17	14/61/28		0.0451 *
		Dominant	CC+CT/TT	56/17	75/28	1.23 (0.6289 to 2.394)	0.5593
		Recessive	CC/CT+TT	21/52	14/89	2.567 (1.239 to 5.255)	0.0130 *
		Allele	C/T	77/69	89/117	1.467 (0.9608 to 2.254)	0.0774
NLRC4	rs212704	Codominant	TT/CT/CC	10/41/22	27/49/27		0.1329
		Dominant	TT+CT/CC	51/22	76/27	0.8236 (0.4285 to 1.578)	0.5672
		Recessive	TT/CT+CC	10/63	27/76	0.4468 (0.2071 to 0.959)	0.0447 *
		Allele	T/C	61/85	103/103	0.7176 (0.4649 to 1.097)	0.1278
NLRC5	rs12598522	Codominant	TT/CT/CC	23/37/13	20/53/30		0.09069
		Dominant	TT+CT/CC	60/13	73/30	1.897 (0.9209 to 3.82)	0.0851
		Recessive	TT/CT+CC	23/50	20/83	1.909 (0.957 to 3.897)	0.0659
		Allele	T/C	83/63	93/113	1.601 (1.048 to 2.47)	0.0305 *
	rs1684579	Codominant	CC/CT/TT	12/31/30	17/60/26		0.0661
		Dominant	CC+CT/TT	43/30	77/26	0.484 (0.2489 to 0.9213)	0.0261 *
		Recessive	CC/CT+TT	12/61	17/86	0.9952 (0.4295 to 2.301)	0.9907
		Allele	C/T	55/91	94/112	0.7201 (0.4725 to 1.111)	0.1364

*, a *p* value < 0.05 indicated a statistically significant difference.

**Table 4:** Genotype distribution of NLRP3 rs3806265, NLRC5 rs1684579 and rs12598522 in non-ABPA group and controls.

Gene	SNP	Model	Non-ABPA Group	Control Group	OR(95% CI)	*p* Value
NLRP3	rs3806265	Codominant	17/24/16	14/61/28		0.0298 *
		Dominant	41/16	75/28	0.9567 (0.4636 to 1.977)	0.9044
		Recessive	17/40	14/89	2.702 (1.172 to 5.924)	0.0129 *
		Allele	58/56	89/117	1.362 (0.8541 to 2.179)	0.1872
NLRC5	rs12598522	Codominant	17/31/9	20/53/30		0.1077
		Dominant	48/9	73/30	2.192 (0.9867 to 5.247)	0.0599
		Recessive	17/40	20/83	1.764 (0.829 to 3.663)	0.1349
		Allele	65/49	93/113	1.612 (1.012 to 2.529)	0.0419 *
	rs1684579	Codominant	9/21/27	17/60/26		0.0125 *
		Dominant	30/27	77/26	0.3752 (0.1889 to 0.7292)	0.0044 **
		Recessive	9/48	17/86	0.9485 (0.4001 to 2.237)	0.9065
		Allele	39/75	94/112	0.6196 (0.381 to 1.006)	0.0471 *

*, a *p* value < 0.05; **, a *p* value < 0.01.

**Table 5:** Genotype distribution of NLRP3 rs3806265 and NLRC5 rs34531240, rs28438857, rs3995818, rs3995817, rs1684579 in IPA group and controls.

Gene	SNP	Model	IPA Group	Control Group	OR(95% CI)	*p* Value
NLRP3	rs3806265	Codominant	13/11/6	14/61/28		0.0017 **
		Dominant	24/6	75/28	1.493 (0.5538 to 3.979)	0.4273
		Recessive	13/17	14/89	4.861 (2.007 to 11.9)	0.0004 ***
		Allele	37/23	89/117	2.115 (1.196 to 3.741)	0.0117 *
NLRC5	rs34531240	Codominant	4/13/13	17/61/25		0.1251
		Dominant	17/13	78/25	0.4191 (0.1796 to 1.026)	0.0420 *
		Recessive	4/26	17/86	0.7783 (0.2655 to 2.484)	0.8928
		Allele	21/39	95/111	0.6291 (0.346 to 1.122)	0.1265
	rs28438857	Codominant	4/13/13	17/61/25		0.1251
		Dominant	17/13	78/25	0.4191 (0.1796 to 1.026)	0.0420 *
		Recessive	4/26	17/86	0.7783 (0.2655 to 2.484)	0.8928
		Allele	21/39	95/111	0.6291 (0.346 to 1.122)	0.1265
	rs3995818	Codominant	4/13/13	17/61/25		0.1251
		Dominant	17/13	78/25	0.4191 (0.1796 to 1.026)	0.0420 *
		Recessive	4/26	17/86	0.7783 (0.2655 to 2.484)	0.8928
		Allele	21/39	95/111	0.6291 (0.346 to 1.122)	0.1265
	rs3995817	Codominant	4/13/13	17/61/25		0.1251
		Dominant	17/13	78/25	0.4191 (0.1796 to 1.026)	0.0420 *
		Recessive	4/26	17/86	0.7783 (0.2655 to 2.484)	0.8928
		Allele	21/39	95/111	0.6291 (0.346 to 1.122)	0.1265
	rs1684579	Codominant	5/9/16	17/60/26		0.0092 **
		Dominant	14/16	77/26	0.2955 (0.1234 to 0.6804)	0.0036 **
		Recessive	5/25	17/86	1.012 (0.3787 to 2.931)	0.9833
		Allele	19/41	94/112	0.5522 (0.3026 to 1)	0.0541

*, a *p* value < 0.05; **, a *p* value < 0.01; ***, a *p* value < 0.001.

**Table 6:** Haplotype analysis for NLRP3 gene polymorphisms in IPA group.

Haplotype	rs34531240	rs28438857	Total	Case	Control	*p* Value
H1	T	C	0.526	0.383	0.568	0.0117 *
H2	C	C	0.289	0.417	0.252	0.0136 *
H3	C	T	0.184	0.200	0.180	0.72

*, a *p* value < 0.05 indicated a statistically significant difference.

## Supplementary Materials

The following supporting information can be downloaded at: https://www.mdpi.com/article/10.3390/jcm11071870/s1, Table S1: Genotype distribution of NLRs SNPs in pulmonary aspergillosis cases and controls; TableS2: Genotype distribution of NLRs SNPs in non-ABPA group, ABPA group and controls; Table S3: Genotype distribution of NLRs SNPs in IPA group, CPA group and controls.
