# Supplementary material for: NLRP3, NLRC4 and NLRC5 Gene Polymorphisms Associate with Susceptibility of Pulmonary Aspergillosis in Non-Neutropenic Patients
Source: J Clin Med. 2022 Mar 28;11(7):1870. doi: 10.3390/jcm11071870 (PMC8999807; doi:10.3390/jcm11071870)
Supplement: Supplementary file 1 [file jcm-11-01870-s001.zip › jcm-1634546-supplementary.pdf]

Supplementary Table S1. Genotype distribution of NLRs SNPs in pulmonary aspergillosis cases and controls.

Gene	SNP	Model	Genotype	Case	Control	OR(95% CI)	*p* Value
NLRP3	rs3806265	Codominant	CC/CT/TT	21/35/17	14/61/28		0.0451 *
		Dominant	CC+CT/TT	56/17	75/28	1.23 (0.6289 to 2.394)	0.5593
		Recessive	CC/CT+TT	21/52	14/89	2.567(1.239 to 5.255)	0.0130 *
		Allele	C/T	77/69	89/117	1.467(0.9608 to 2.254)	0.0774
	rs7525979	Codominant	TT/CT/CC	3/23/47	0/37/66		0.1066
		Dominant	TT+CT/CC	26/47	37/66	0.9868(0.5271 to 1.807)	0.9667
		Recessive	TT/CT+CC	3/70	0/103	Infinity(1.241 to Infinity)	0.1378
		Allele	T/C	29/117	37/169	1.132(0.652 to 1.917)	0.6524
	rs10754558	Codominant	GG/CG/CC	20/28/25	26/45/32		0.7780
		Dominant	GG+CG/CC	48/25	71/32	0.8654(0.4591 to 1.662)	0.6571
		Recessive	GG/CG+CC	20/53	26/77	1.118(0.562 to 2.153)	0.7486
		Allele	G/C	68/78	97/109	0.9796(0.6403 to 1.495)	0.9244
NLRC4	rs12989936	Codominant	TT/CT/CC	6/34/33	14/48/41		0.5012
		Dominant	TT+CT/CC	40/33	62/41	0.8016(0.4286 to 1.501)	0.4746
		Recessive	TT/CT+CC	6/67	14/89	0.5693(0.2109 to 1.473)	0.2685
		Allele	T/C	46/100	76/130	0.7868(0.4971 to 1.221)	0.2954
	rs212704	Codominant	TT/CT/CC	10/41/22	27/49/27		0.1329
		Dominant	TT+CT/CC	51/22	76/27	0.8236(0.4285 to 1.578)	0.5672
		Recessive	TT/CT+CC	10/63	27/76	0.4468(0.2071 to 0.959)	0.0447 *
		Allele	T/C	61/85	103/103	0.7176(0.4649 to 1.097)	0.1278
	rs7562653	Codominant	CC/CT/TT	14/32/27	16/54/33		0.5260
		Dominant	CC+CT/TT	46/27	70/33	0.8032(0.4323 to 1.508)	0.4951
		Recessive	CC/CT+TT	14/59	16/87	1.29(0.6008 to 2.808)	0.5265
		Allele	C/T	60/86	86/120	0.9735(0.6276 to 1.5)	0.9027
	rs479333	Codominant	GG/CG/CC	6/36/31	13/47/43		0.6392
		Dominant	GG+CG/CC	42/31	60/43	0.971(0.521 to 1.743)	0.9242
		Recessive	GG/CG+CC	6/67	13/90	0.62(0.2264 to 1.655)	0.3538
		Allele	G/C	48/98	73/133	0.8924(0.5667 to 1.384)	0.6183
	rs385076	Codominant	CC/CT/TT	12/33/28	15/51/37		0.8468
		Dominant	CC+CT/TT	45/28	66/37	0.901(0.4956 to 1.659)	0.7417
		Recessive	CC/CT+TT	12/61	15/88	1.154(0.4848 to 2.567)	0.7338
		Allele	C/T	57/89	81/125	0.9883(0.6479 to 1.534)	0.9578
NLRC5	rs12598522	Codominant	TT/CT/CC	23/37/13	20/53/30		0.09069
		Dominant	TT+CT/CC	60/13	73/30	1.897(0.9209 to 3.82)	0.0851
		Recessive	TT/CT+CC	23/50	20/83	1.909(0.957 to 3.897)	0.0659
		Allele	T/C	83/63	93/113	1.601(1.048 to 2.47)	0.0305 *
	rs34531240	Codominant	TT/CT/CC	13/34/26	17/61/25		0.2013
		Dominant	TT+CT/CC	47/26	78/25	0.5794(0.3094 to 1.142)	0.1022
		Recessive	TT/CT+CC	13/60	17/86	1.096(0.4972 to 2.48)	0.8208
		Allele	T/C	60/86	95/111	0.8152(0.527 to 1.25)	0.3499
	rs28438857	Codominant	TT/CT/CC	13/34/26	17/61/25		0.2013
		Dominant	TT+CT/CC	47/26	78/25	0.5794(0.3094 to 1.142)	0.1022
		Recessive	TT/CT+CC	13/60	17/86	1.096(0.4972 to 2.48)	0.8208
		Allele	T/C	60/86	95/111	0.8152(0.527 to 1.25)	0.3499
	rs3995818	Codominant	AA/AG/GG	13/35/25	17/61/25		0.2815
		Dominant	AA+AG/GG	48/25	78/25	0.6154(0.3271 to 1.226)	0.1483
		Recessive	AA/AG+GG	13/60	17/86	1.096(0.4972 to 2.48)	0.8208
		Allele	A/G	61/85	95/111	0.8385(0.543 to 1.284)	0.4198

rs3995817	Codominant	CC/CT/TT	13/34/26	17/61/25		0.2013
	Dominant	CC+CT/TT	47/26	78/25	0.5794(0.3094 to 1.142)	0.1022
	Recessive	CC/CT+TT	13/60	17/86	1.096(0.4972 to 2.48)	0.8208
	Allele	C/T	60/86	95/111	0.8152(0.527 to 1.25)	0.3499
rs1684579	Codominant	CC/CT/TT	12/31/30	17/60/26		0.0661
	Dominant	CC+CT/TT	43/30	77/26	0.484(0.2489 to 0.9213)	0.0261 *
	Recessive	CC/CT+TT	12/61	17/86	0.9952(0.4295 to 2.301)	0.9907
	Allele	C/T	55/91	94/112	0.7201(0.4725 to 1.111)	0.1364
rs3751705	Codominant	CC/CT/TT	9/32/32	12/41/50		0.8241
	Dominant	CC+CT/TT	41/32	53/50	1.209(0.6563 to 2.201)	0.5373
	Recessive	CC/CT+TT	9/64	12/91	1.066(0.4473 to 2.569)	0.8912
	Allele	C/T	50/96	65/141	1.13(0.7159 to 1.767)	0.5956

*, a p value < 0.05 indicated a statistically significant difference.

Supplementary Table S2. Genotype distribution of NLRs SNPs in non-ABPA group, ABPA group and controls.

Gene	SNP	Model	Non-ABPA Group	ABPA Group	Control Group	P1 Value [1]	P2 Value [2]
NLRP3	rs3806265	Codominant	17/24/16	4/11/1	14/61/28	0.0298 *	0.1430
		Dominant	41/16	15/1	75/28	0.9044	0.1140
		Recessive	17/40	4/12	14/89	0.0129 *	0.4180
		Allele	58/56	19/13	89/117	0.1872	0.0874
	rs7525979	Codominant	3/17/37	0/6/10	0/37/66	0.0542	NA
		Dominant	20/37	6/10	37/66	0.9159	0.9027
		Recessive	3/54	0/16	0/103	0.0815	NA
		Allele	23/91	6/26	37/169	0.6270	0.9141
	rs10754558	Codominant	18/21/18	2/7/7	26/45/32	0.6201	0.4415
		Dominant	39/18	9/7	71/32	0.9468	0.3147
		Recessive	18/39	2/14	26/77	0.3900	0.4230
		Allele	57/57	11/21	97/109	0.6175	0.1790
NLRC4	rs12989936	Codominant	4/26/27	2/8/6	14/48/41	0.3845	0.9681
		Dominant	30/27	10/6	62/41	0.3541	0.8607
		Recessive	4/53	2/14	14/89	0.3177	0.7835
		Allele	34/80	12/20	76/130	0.2023	0.9473
	rs212704	Codominant	8/31/18	2/10/4	27/49/27	0.2015	0.4267
		Dominant	39/18	12/4	76/27	0.4698	0.8390
		Recessive	8/49	2/14	27/76	0.0743	0.3812
		Allele	47/67	14/18	103/103	0.1321	0.5106
	rs7562653	Codominant	9/26/22	5/6/5	16/54/33	0.6699	0.2790
		Dominant	35/22	11/5	70/33	0.4030	0.9498
		Recessive	9/48	5/11	16/87	0.9660	0.1250
		Allele	44/70	16/16	86/120	0.5826	0.3801
	rs479333	Codominant	4/27/26	2/9/5	13/47/43	0.5391	0.7010
		Dominant	31/26	11/5	60/43	0.6363	0.4258
		Recessive	4/53	2/14	13/90	0.4044	0.6956
		Allele	35/79	13/19	73/133	0.3910	0.5698
	rs385076	Codominant	8/26/23	4/7/5	15/51/37	0.8543	0.5698
		Dominant	34/23	11/5	66/37	0.5795	0.7160
		Recessive	8/49	4/12	15/88	0.9274	0.4880

		Allele	42/72	15/17	81/125	0.6625	0.4177
NLRC5	rs12598522	Codominant	17/31/9	6/6/4	20/53/30	0.1077	0.2594
		Dominant	48/9	12/4	73/30	0.0599	0.9661
		Recessive	17/40	6/10	20/83	0.1349	0.1034
		Allele	65/49	18/14	93/113	0.0419 *	0.2414
	rs34531240	Codominant	9/26/22	4/8/4	17/61/25	0.1477	0.6796
		Dominant	35/22	12/4	78/25	0.0568	0.8027
		Recessive	9/48	4/12	17/86	0.9065	0.6335
		Allele	44/70	16/16	95/111	0.1937	0.6820
	rs28438857	Codominant	9/26/22	4/8/4	17/61/25	0.1477	0.6796
		Dominant	35/22	12/4	78/25	0.0568	0.8027
		Recessive	9/48	4/12	17/86	0.9065	0.6335
		Allele	44/70	16/16	95/111	0.1937	0.6820
	rs3995818	Codominant	9/27/21	4/8/4	17/61/25	0.2270	0.6796
		Dominant	36/21	12/4	78/25	0.0925	0.8027
		Recessive	9/48	4/12	17/86	0.9065	0.6335
		Allele	45/69	16/16	95/111	0.2513	0.6820
	rs3995817	Codominant	9/26/22	4/8/4	17/61/25	0.1477	0.6796
		Dominant	35/22	12/4	78/25	0.0568	0.8027
		Recessive	9/48	4/12	17/86	0.9065	0.6335
		Allele	44/70	16/16	95/111	0.1937	0.6820
	rs1684579	Codominant	9/21/27	3/10/3	17/60/26	0.0125 *	0.8506
		Dominant	30/27	13/3	77/26	0.0044 **	0.8027
		Recessive	9/48	3/13	17/86	0.9065	0.8919
		Allele	39/75	16/16	94/112	0.0471 *	0.6447
	rs3751705	Codominant	8/24/25	1/8/7	12/41/50	0.8243	0.6751
		Dominant	32/25	9/7	53/50	0.5696	0.7210
		Recessive	8/49	1/15	12/91	0.6623	1
		Allele	40/74	10/22	65/141	0.5190	0.9726

[1] P1: The association between NLR genes polymorphisms and non-ABPA.

[2] P2: The association between NLR genes polymorphisms and ABPA

*, a p value < 0.05; **, a p value < 0.01.

Supplementary Table S3. Genotype distribution of NLRs SNPs in IPA group, CPA group and controls.

Gene	SNP	Model	IPA Group	CPA Group	Control Group	P1 Value [1]	P2 Value [2]
NLRP3	rs3806265	Codominant	13/11/6	4/13/10	14/61/28	0.0017 **	0.5503
		Dominant	24/6	17/10	75/28	0.4273	0.3164
		Recessive	13/17	4/23	14/89	0.0004 ***	0.8813
		Allele	37/23	21/33	89/117	0.0117 *	0.5678
	rs7525979	Codominant	1/10/19	2/7/18	0/37/66	0.1756	0.0159
		Dominant	11/19	9/18	37/66	0.9405	0.8023
		Recessive	1/29	2/25	0/103	0.2256	0.0419
		Allele	12/48	11/43	37/169	0.7200	0.6846
	rs10754558	Codominant	6/12/12	12/9/6	26/45/32	0.6378	0.1478
		Dominant	18/12	21/6	71/32	0.3602	0.3684
		Recessive	6/24	12/15	26/77	0.5544	0.0509
		Allele	24/36	33/21	97/109	0.3319	0.0666

NLRC4	rs12989936	Codominant	2/14/14	2/12/13	14/48/41	0.5514	0.5931
		Dominant	16/14	14/13	62/41	0.5019	0.4336
		Recessive	2/28	2/25	14/89	0.4794	0.5880
		Allele	18/42	16/38	76/130	0.3256	0.3204
	rs212704	Codominant	4/16/10	4/15/8	27/49/27	0.3271	0.4641
		Dominant	20/10	19/8	76/27	0.4438	0.7217
		Recessive	4/26	4/23	27/76	0.2213	0.3254
		Allele	24/36	23/31	103/103	0.1723	0.3323
	rs7562653	Codominant	5/13/12	4/13/10	16/54/33	0.6585	0.8848
		Dominant	18/12	17/10	70/33	0.4174	0.6232
		Recessive	5/25	4/23	16/87	0.8810	0.8357
		Allele	23/37	21/33	86/120	0.6360	0.7040
	rs479333	Codominant	2/14/14	2/13/12	13/47/43	0.6482	0.7520
		Dominant	16/14	15/12	60/43	0.6319	0.8007
		Recessive	2/28	2/25	13/90	0.5794	0.6771
		Allele	18/42	17/37	73/133	0.4347	0.5866
	rs385076	Codominant	4/14/12	4/12/11	15/51/37	0.9194	0.8830
		Dominant	18/12	16/11	66/37	0.6837	0.6442
		Recessive	4/26	4/23	15/88	0.8989	0.7848
		Allele	22/38	20/34	81/125	0.7104	0.7593
NLRC5	rs12598522	Codominant	8/17/5	9/14/4	20/53/30	0.3552	0.1709
		Dominant	25/5	23/4	73/30	0.1726	0.2076
		Recessive	8/22	9/18	20/83	0.3914	0.1221
		Allele	33/27	32/22	93/113	0.1785	0.0646
	rs34531240	Codominant	4/13/13	5/13/9	17/61/25	0.1251	0.5533
		Dominant	17/13	18/9	78/25	0.0420 *	0.3403
		Recessive	4/26	5/22	17/86	0.8928	0.8038
		Allele	21/39	23/31	95/111	0.1265	0.6434
	rs28438857	Codominant	4/13/13	5/13/9	17/61/25	0.1251	0.5533
		Dominant	17/13	18/9	78/25	0.0420 *	0.3403
		Recessive	4/26	5/22	17/86	0.8928	0.8038
		Allele	21/39	23/31	95/111	0.1265	0.6434
	rs3995818	Codominant	4/13/13	5/14/8	17/61/25	0.1251	0.7809
		Dominant	17/13	19/8	78/25	0.0420 *	0.5691
		Recessive	4/26	5/22	17/86	0.8928	0.8038
		Allele	21/39	24/30	95/111	0.1265	0.8262
	rs3995817	Codominant	4/13/13	5/13/9	17/61/25	0.1251	0.5533
		Dominant	17/13	18/9	78/25	0.0420 *	0.3403
		Recessive	4/26	5/22	17/86	0.8928	0.8038
		Allele	21/39	23/31	95/111	0.1265	0.6434
	rs1684579	Codominant	5/9/16	4/12/11	17/60/26	0.0092 **	0.2753
		Dominant	14/16	16/11	77/26	0.0036 **	0.1122
		Recessive	5/25	4/23	17/86	0.9833	0.9352
		Allele	19/41	20/34	94/112	0.0541	0.2573
	rs3751705	Codominant	4/14/12	4/10/13	12/41/50	0.7107	0.8974
		Dominant	18/12	14/13	53/50	0.4091	0.9708
		Recessive	4/26	4/23	12/91	0.9446	0.9073
		Allele	22/38	18/36	65/141	0.4575	0.8028

[1]P1: The association between NLR genes polymorphisms and IPA

[2]P2: The association between NLR genes polymorphisms and CPA

*, a p value < 0.05; **, a p value <0.01; ***, a p value <0.001.
